# Insights into the Technological Evolution and Research Trends of Mobile Health: Bibliometric Analysis

**DOI:** 10.3390/healthcare13070740

**Published:** 2025-03-26

**Authors:** Ruichen Zhang, Hongyun Wang

**Affiliations:** 1 SILC Business School, Shanghai University, Shanghai 200444, China; 13681952896@shu.edu.cn; 2 Cardiac Regeneration and Ageing Lab, Institute of Geriatrics (Shanghai University), Affiliated Nantong Hospital of Shanghai University (The Sixth People’s Hospital of Nantong), School of Life Science, Shanghai University, Nantong 226011, China; 3 Shanghai Engineering Research Center of Organ Repair, School of Life Science, Shanghai University, Shanghai 200444, China; 4 Joint International Research Laboratory of Biomaterials and Biotechnology in Organ Repair (Ministry of Education), School of Life Science, Shanghai University, Shanghai 200444, China

**Keywords:** mobile health (mHealth), mHealth app, mental health, digital intervention, bibliometric analysis, data visualization

## Abstract

**Background/Objectives**: Smartphones, with their widespread popularity and diverse apps, have become essential in our daily lives, and ongoing advancements in information technology have unlocked their significant potential in healthcare. Our goal is to identify the future research directions of mobile health (mHealth) by examining its research trends and emerging hotspots. **Methods**: This study collected mHealth-related literature published between 2005 and 2024 from the Web of Science database. We conducted a descriptive statistic of the annual publication count and categorized the data by authors and institutions. In addition, we developed visualization maps to display the frequency of keyword co-occurrences. Furthermore, overlay visualizations were created to showcase the average publication year of specific keywords, helping to track the changing trends in mHealth research over time. **Results**: Between 2005 and 2024, a total of 6093 research papers related to mHealth were published. The data have revealed a rapid increase in the number of publications since 2011. However, it was found that research on mHealth has reached a saturation point since 2021. The University of California was the dominant force in mHealth research, with 248 articles. The University of California, the University of London, Harvard University, and Duke University are actively collaborating, which shows a geographical pattern of collaboration. From the analysis of keyword co-occurrence and timeline, the research focus has gradually shifted from solely mHealth technologies to exploring how new technologies, such as artificial intelligence (AI) in mobile apps, can actively intervene in patient conditions, including breast cancer, diabetes, and other chronic diseases. Privacy protection policies and transparency mechanisms have emerged as an active research focus in current mHealth development. Notably, cutting-edge technologies such as the Internet of Things (IoT), blockchain, and virtual reality (VR) are being increasingly integrated into mHealth systems. These technological convergences are likely to constitute key research priorities in the field, particularly in addressing security vulnerabilities while enhancing service scalability. **Conclusions**: Although the volume of core research in mobile health (mHealth) is gradually declining, its practical applications continue to expand across diverse domains, increasingly integrating with multiple emerging technologies. It is believed that mobile health still holds enormous potential.

## 1. Introduction

Mobile health (mHealth), introduced in 2007 as a subset of electronic health (ehealth), refers to healthcare services supported by mobile communication devices such as smartphones, wireless patient monitoring devices, and tablets [1,2]. By 2020, mHealth encompassed medical and public health practices utilizing these mobile technologies [3]. Since mHealth is a relatively broad concept, in this article, mHealth mainly refers to the provision of medical services through mobile phones or other wireless devices.

Progress in mHealth over the last decade has been unprecedented. This tremendous progress has made it possible to foresee the emergence of new digital trends on a global scale. mHealth is now the basis for numerous successful applications in many healthcare areas including disease prediction, prevention, management, and diagnostics. It also shows the improvements in treatments, patient education, and empowerment [4].

The proliferation of mobile technology has revolutionized healthcare delivery, with the increasing adoption of mobile medical services offering new opportunities for information technology applications in healthcare. A previous study demonstrated WeChat-based telemedicine for COVID-19 home monitoring. Another study highlighted that user participation is important in mHealth development in Germany. Moreover, a previous study established frameworks for evaluating mHealth app quality and effectiveness [5,6,7]. Unlike traditional medical resources, which are limited by location and accessibility constraints [8,9], mobile internet technology and smartphones provide flexible, effective healthcare delivery methods. Currently, over 100,000 mHealth applications are available on major app stores, most at minimal or no cost, featuring advanced sensor integration and sophisticated functions [10]. The mHealth market is projected to grow at 17.6% annually from 2021 to 2028 [11]. This expansion is particularly valuable in developing countries with limited clinical resources. Recognizing these benefits, the World Health Organization has endorsed mHealth as a critical tool for achieving sustainable development goals through cost-effective, convenient, and transparent healthcare access [12].

Bibliometric analysis has gained popularity across disciplines for its flexibility and efficiency. At this critical stage in mHealth’s development, it offers strategic insights unavailable through conventional reviews [13]. As mHealth rapidly expands, bibliometric approaches help stakeholders navigate its complexity by quantitatively mapping research trends, collaboration networks, and knowledge diffusion patterns. This methodology identifies research gaps, prevents duplication, and optimizes resource allocation during mHealth’s transition from innovation to implementation. It informs evidence-based decisions by revealing promising technologies, effective clinical applications, and high-impact collaborations. Bibliometric insights can guide targeted implementation strategies by identifying proven, contextually appropriate solutions. Therefore, to understand this evolving field, we conducted a bibliometric analysis examining past and present mHealth developments, aiming to provide fresh perspectives on future research directions.

## 2. Materials and Methods

Before the literature retrieval and analysis, we conducted a systematic research design planning phase to ensure maximum capture of the relevant literature in the mobile health field. The keyword selection underwent multiple rounds of discussion; we chose “Mobile Health”, “mHealth”, and “Mobile Medical” as primary concepts because they are the most used and widely recognized terms in the field. Simultaneously, we combined these terms with mobile device-related vocabulary (such as “Mobile Devices”, “Mobile Technology”, etc.) and healthcare domain terminology (such as “Healthcare”, “Medicine”, etc.), forming a comprehensive search strategy to ensure coverage of multidimensional research in the field. This multi-level keyword combination aimed to balance search sensitivity and specificity, avoiding omission of important literature while reducing irrelevant results.

Methodologically, we employed a mixed-method design, combining quantitative bibliometric analysis with qualitative thematic analysis. The selection of CiteSpace 6.4.R1 and VOSviewer 1.6.20 as analytical tools was based on their complementary strengths in visualizing scientific knowledge maps, identifying research frontiers, and evolution paths: CiteSpace excels in displaying knowledge evolution processes across the temporal dimension, while VOSviewer performs exceptionally in revealing relationship networks between research topics [14,15]. This complementary tool combination provided us with the capability to analyze the mobile health research ecosystem from multiple perspectives.

The literature is extracted from the Science Citation Index Expanded and Social Science Citation Index of the Web of Science Core Collection. The search period spans from January 2005 to December 2024. The keywords for mHealth care are TS = (“Mobile Health” OR “mHealth” OR “Mobile Medical”) AND TS = (“Mobile Devices” OR “Mobile Technology” OR “Mobile Communication” OR “Wireless Devices” OR “Wireless Technology” OR “Wireless Applications” OR “Communication Technology” OR “Applications” OR “App”) AND TS = (“Healthcare” OR “Health” OR “Medicine” OR “Hospital” OR “Nursing”). Selected articles from January 2005 to December 2024, excluding conference abstracts, editorial materials, and online publications, a total of 6093 records of articles and review articles were kept. CiteSpace 6.4.R1 and VOSviewer 1.6.20 were used to comb and visualize the data of the included 6093 valid citations in our prior study.

Our inclusion criteria focused on peer-reviewed research articles and reviews explicitly addressing mHealth technologies (2005–2024) from journals indexed in Science Citation Index Expanded and Social Science Citation Index. We excluded conference abstracts due to their limited methodological detail and preliminary nature, editorial materials as they represent opinions rather than original research, and online-only publications without formal journal assignments to ensure data consistency. To address potential database selection, we conducted supplementary searches using similar search terms in PubMed and Google Scholar databases as reference comparisons. This systematic approach to the literature selection allowed us to comprehensively analyze the development and current state of mobile health research while minimizing selection biases through cross-database verification of major findings and trends.

**Figure 1:**
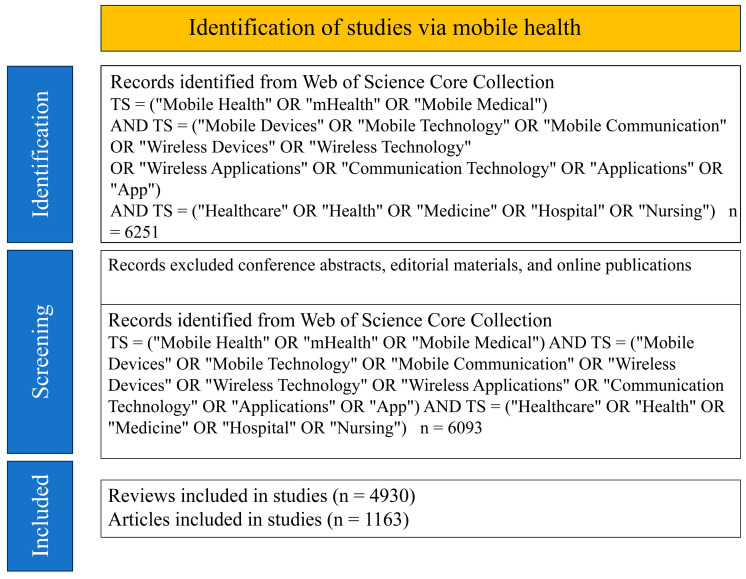
Flowchart of the literature selection all the data were collected from Web of Science core collection. Notes. TS: topic search.

Finally, we drew the PRISMA chart based on a template [16] to depict the literature selection process (Figure 1).

## 3. Results

### 3.1. Analysis of the Trend of Publications

**Figure 2:**
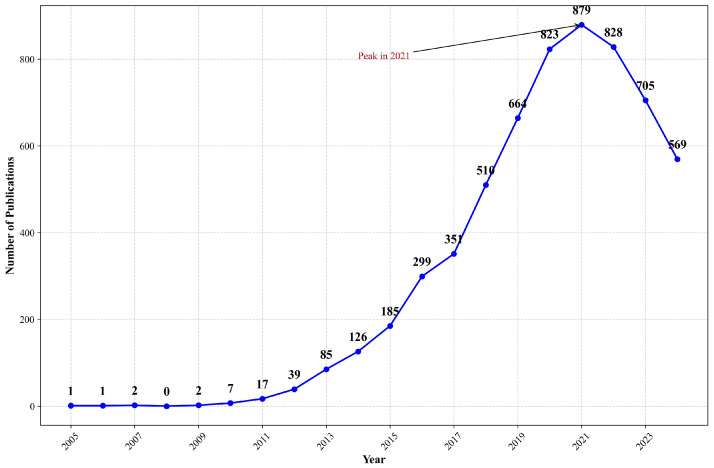
Number of publications about mHealth from 2005 to 2024 in WOS. Note: This figure shows the annual publication counts related to mobile health (mHealth) indexed in Web of Science from 2005 to 2024. The data reveals significant growth since 2010, with acceleration after 2015, corresponding to smartphone proliferation and digital health advances.

Due to the fact that articles for 2024 were still being published during the time of this analysis, we primarily focused on the publication data from 2005 to 2023 (File S1). From 2005 to 2023, the number of studies related to mHealth has generally shown an upward trend. Before 2011, there were few articles on mHealth. The period from 2011 to 2015 was a slow growth phase, followed by a period of rapid development from 2015 to 2020. After 2020, the enthusiasm seems to have waned (Figure 2).

**Figure 3:**
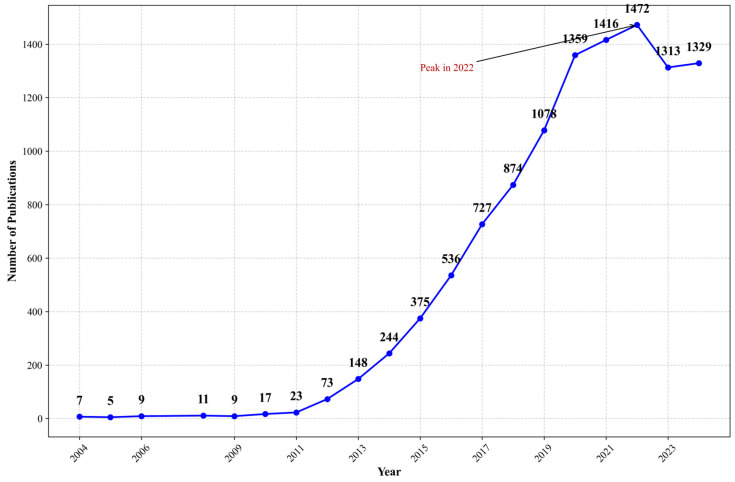
Number of publications about mHealth from 2005 to 2024 in PubMed. Note: This figure shows publications on mHealth in PubMed which shows potential growth from 2005 (<10 publications/year) to 2022 (peak of 1472 publications). After 2022, publication numbers slightly decreased but remained high (>1300/year).

**Figure 4:**
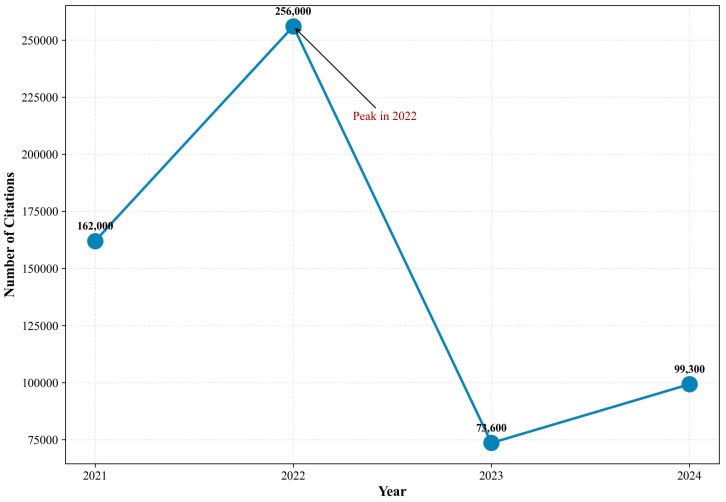
Number of publications about mHealth from 2021 to 2024 in Google Scholar. Note: This figure displays the number of mHealth publications in Google Scholar from 2021 to 2024. Publications increased dramatically from 162,900 in 2021 to a peak of 256,900 in 2022. After 2022, there was a sharp decline to 73,600 publications in 2023, followed by a moderate recovery to 99,300 in 2024.

According to the retrieval results from PubMed and Google Scholar (Figure 3 and Figure 4), publication trends differ due to the varying search mechanisms employed by WOS, PubMed, and Google Scholar (File S1). Notable differences in publication trends are evident: both databases show significant growth from 2012 to 2020, with peaks occurring in 2022, though the subsequent decline patterns vary considerably. This variation likely stems from PubMed’s focus on biomedical literature and clinical studies, while Google Scholar encompasses a broader range of resources, including gray literature and preprints. These findings suggest that while core digital research may be decreasing, peripheral research continues to expand. This pattern indicates a potential shift in research focus from basic concept validation to advanced applications, suggesting the field has matured and entered an integration phase.

**Figure 5:**
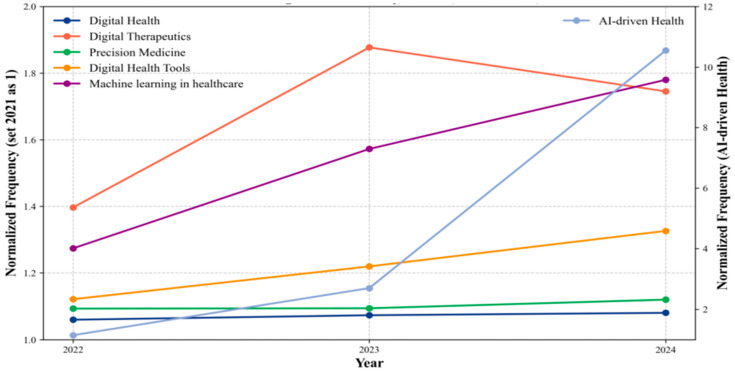
Trend in Digital Health Keywords from 2022 to 2024. Note: Digital health trends (2022–2024) show Digital Diagnostics surging early and then plateauing, while AI-driven Health dramatically increased in the final year. Machine learning in healthcare grew steadily throughout. Precision Medicine showed modest growth, while Digital Health and Digital Health Tools remained relatively unchanged. This suggests a shift from diagnostic technologies toward AI applications in healthcare.

The trend of interest in both telehealth and wireless health is on the decline (File S1). It suggests a general waning in the mHealth sector. We noticed that within the broader digital health field, some other hotspot keywords have shown an increase (Figure 5). This may suggest a shift in focus from mobile health to other areas within the digital health domain. Digital health has seen a stable change from 2021 to 2023, which reflects its expanding role in healthcare systems globally. Similarly, precision medicine has shown steady increases. It indicates the continued emphasis on personalized treatments based on genetic, environmental, and lifestyle factors. AI-driven health and machining learning in healthcare have experienced a remarkable surge, particularly in 2023, highlighting the growing influence of artificial intelligence (AI) in healthcare for diagnostics, personalized medicine, and operational improvements. On the other hand, digital therapeutics experienced an initial rise but showed a slight decrease in 2023. It shows a maturing field with challenges in widespread adoption. These trends underscore the continued transformation of healthcare through technological advancements, with AI, digital therapeutics, and precision medicine at the forefront of this evolution.

Besides, we also analyzed the overall situation of Journals and Co-Cited Academic Journals, Authors and Co-cited authors. However, due to space limitations, we placed them in the appendix (Appendix A and Appendix B).

### 3.2. Distribution of Institutions

**Figure 6:**
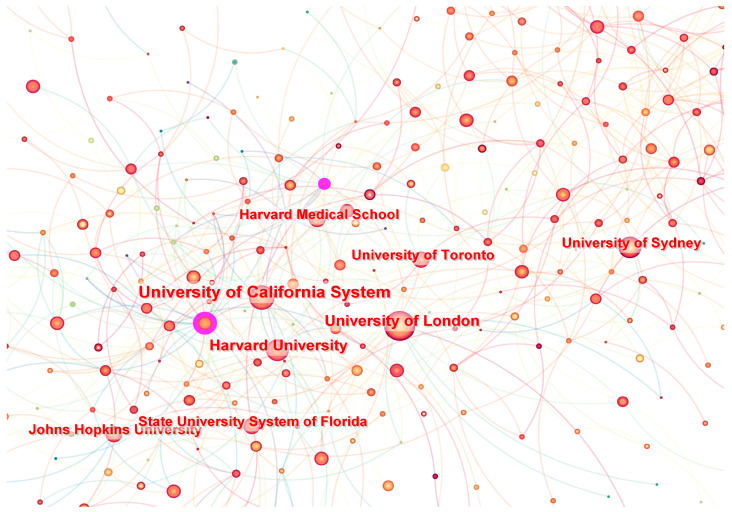
Networks of publications from different institutions about mHealth. Note: Node size represents academic influence, color indicates active years (red for 2011, yellow for 2021), edge thickness reflects collaboration strength, and purple rings highlight high-centrality institutions.

A total of 525 institutions have made contributions to research on mHealth. To better understand the distribution of the author’s countries and institutions, we constructed a map of these articles via Citespace. The visualization analysis diagram shows 525 nodes and 828 connections, with a network density of 0.0064 (Figure 6). It is a visual analysis of the aforementioned results through CiteSpace, where each node represents an institution, and the size of the node is proportional to the number of articles published. The University of California, the University of London, Harvard University, and Duke University are actively collaborating. Within these entities, the University of California leads in publication volume with 248 articles, followed by the University of London and Harvard University, both of which have published over 200 articles (Table 1). Moreover, IDIBAPS in Spain has the highest centrality of 0.09 in the field of mHealth research. This indicates that it holds a significant level of importance or influence within the network. As a result, the research on mHealth is primarily concentrated in European and American research institutions.

### 3.3. Analysis of Keywords

**Figure 7:**
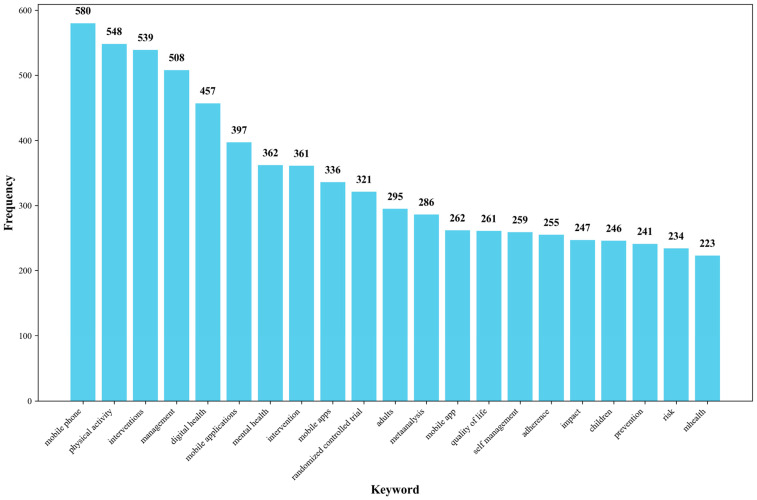
The top 20 high-frequency keywords in the field of mHealth research. Note: Figure shows the top 20 keywords in mHealth research. Mobile health leads with 580 occurrences, followed by health (548), intervention (539), smartphone (508), and mobile application (457). This distribution highlights the field’s focus on mobile technologies for health interventions, with technical and implementation aspects as key research priorities.

In the keyword section, terms with broad usage such as “mHealth”, “technology”, “health”, and “care” have been excluded. Figure 7 presents the top 20 most frequent keywords (File S1). Among these keywords, “mobile phone” (580 counts) and “mobile app” (336 counts) appear multiple times, indicating that mHealth still primarily relies on smartphones as the main medium [17]. The frequent occurrence of “physical activity” (548 counts) highlights its role as a primary health intervention. The terms “adults” (311 counts) and “children” (246 counts) suggest that the main target populations for mHealth initiatives are likely adults and children. The presence of “metaanalysis” (286 counts) and “randomized controlled trial” (321 counts) signifies the research methodologies employed in the field of mHealth. Keywords like “interventions”(361 counts), “management” (508 counts) and “self-management” (259 counts) primarily point to the focus of mHealth research on health interventions and management [17]. The inclusion of “mental health” (362 counts) emphasizes the importance of mental health in the domain of mHealth [18].

**Figure 8:**
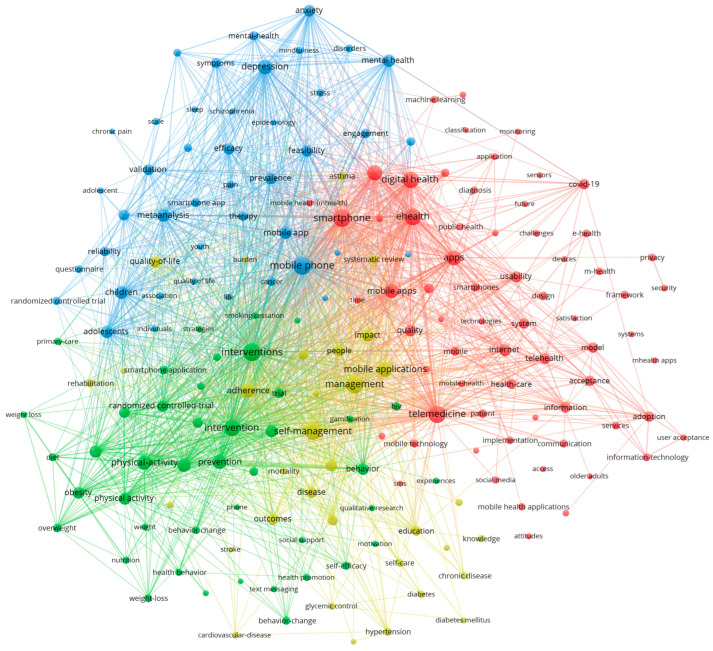
Keyword Clustering in mHealth Research Visualized Using VOSviewer. Note: Node colors represent different thematic clusters labeled with their dominant topics. This network visualization maps the digital health research landscape, showing distinct clusters: mental health (blue), digital technologies (red), lifestyle interventions (green), and chronic disease management (yellow). Key bridging concepts include smartphones and mobile applications.

The VOSviewer software was used to cluster main keywords (counts ≥ 50) in the field of mHealth (Figure 8). Circles and labels form individual elements, with the color of an element indicating the cluster it belongs to. The analysis revealed four research directions represented by red, blue, yellow, and green clusters. The red cluster represents key concepts in the area, such as digital health, ehealth, and medicine. The green cluster primarily addresses technical issues like information systems, medical safety, algorithms, and environmental factors. The blue cluster focuses on mental health and the target population, emphasizing the application of mHealth. The yellow cluster highlights mobile apps and management, showcasing the carriers and practical applications of mHealth. These themes provide a comprehensive summary of the research perspectives on mHealth.

**Figure 9:**
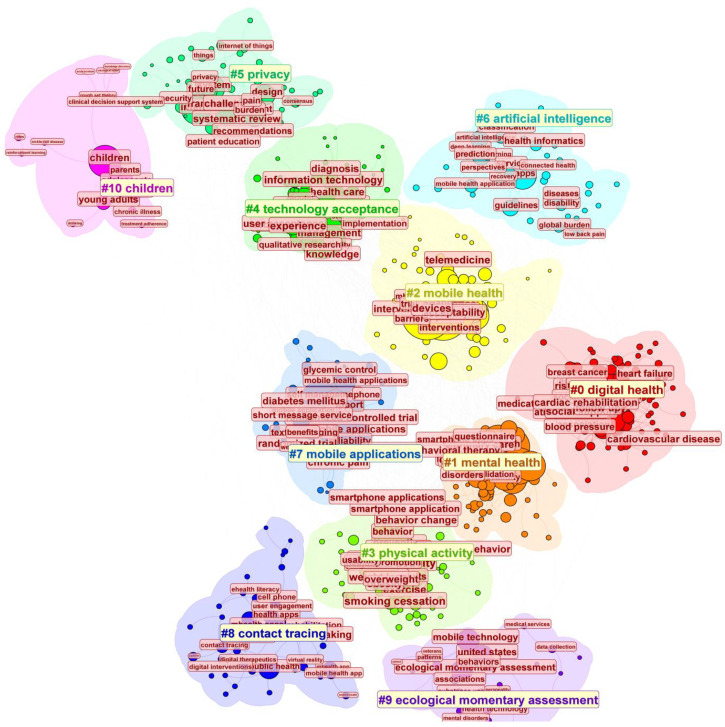
Keyword Clustering Analysis of mHealth Research Using CiteSpace. Note: This visualization presents a thematic clustering of digital health research areas, organized into 11 distinct clusters identified by numbers (#0–#10). Each cluster represents a key research domain: children’s health (pink, **top left**), privacy concerns (light green, **top**), artificial intelligence applications (light blue, **top right**), technology acceptance (green, **top**), mobile health and telemedicine (yellow, **center right**), digital health interventions (red, **center right**), mobile applications (light blue, **center left**), physical activity (light green, **bottom center**), mental health (orange, **center**), contact tracing (deep blue, **bottom left**), and ecological momentary assessment methods (purple, **bottom**).

In detail, the CiteSpace analysis of keyword clusters in mHealth research identified ten key themes (Figure 9). The modularity value (Q = 0.4192) indicates a good quality of division, with distinct and independent clusters representing different research themes. The relatively high Q value suggests that keywords within each cluster are strongly connected, while connections between clusters are weaker. This demonstrates that the clustering effectively captures the structure and boundaries of the research topics. Digital Health, the largest theme, reflects the integration of technology into healthcare to help with various illnesses like breast cancer and high blood pressure [19,20].

The second largest theme, mental health focuses on using mobile tools for psychological well-being, including cognitive behavioral therapy and mindfulness apps [21]. Mobile application, which is near the theme of mental health, represents studies centered on the use of smartphone apps for health interventions, highlighting design, functionality, and user engagement. The overlap of smartphone apps and mental health highlights the integration of mobile apps in managing both psychological support and chronic diseases like diabetes, reflecting a trend toward holistic, multifunctional health solutions. Under the mobile application, physical activity highlights how mHealth technologies are being applied to a variety of health challenges. Physical activity explores the role of apps and wearable devices in promoting exercise and healthier lifestyles. In summary, the convergence of mobile applications in mental health and physical activity underscores a shift toward integrated, multifunctional health solutions, where technology plays a pivotal role in supporting both psychological well-being and physical health, fostering a more comprehensive approach to overall wellness.

The categories at the top of the image represent emerging directions. Artificial intelligence (AI) advances innovations in diagnostics, predictive modeling, and personalized care [22]. The theme of children may mean that mHealth addresses youth-specific health issues, such as mental health support and chronic disease management. Technology acceptance examines factors influencing the acceptance and integration of mHealth technologies, highlighting mobile solutions for diagnosis, treatment monitoring, and support. However, the diverse perspectives and concerns of patients regarding the privacy, security, and confidentiality of data in mHealth apps also appear [23]. In conclusion, these emerging directions emphasize the transformative role of mobile health technologies in addressing new health challenges and improving the acceptance of AI-driven healthcare solutions.

### 3.4. Timeline Analysis of Keywords

Timeline visualization is a method for visualizing research hotspots and their clustering relationships over time by using time slices. It provides an intuitive view of the chronological appearance and development of research hotspots along the time axis.

**Figure 10:**
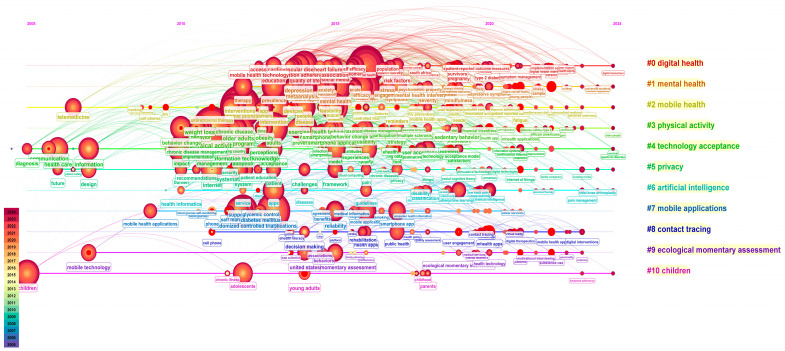
Timeline of Research Trends and Clusters in mHealth: 2005–2024. Note: This horizontal flow visualization displays the digital health research landscape as a timeline progression, with 11 color-coded thematic streams (#0–#10) running left to right. The red nodes represent research publications, with node size indicating citation impact. Key themes include digital health (red), mental health (orange), mobile health (yellow), physical activity (light green), technology acceptance (green), privacy (dark green), artificial intelligence (light blue), mobile applications (blue), contact tracing (dark blue), ecological momentary assessment (deep purple) and children (pink).

This timeline consists of 10 clusters (Figure 10), with digital health being the most active area of research. Before 2010, the number of keywords was relatively limited, with only a few key terms such as “telemedicine”, “healthcare”, “information”, and “children” being commonly used. This reflects a time when mHealth and related technologies were still emerging, and the focus was primarily on foundational concepts like mobile health technology. From 2011 to 2022, with the growth of wireless devices, it focused on various aspects of health monitoring and disease prevention based on mobile devices and remained active. During the period from 2011 to 2015, there was an explosive growth in keywords related to mHealth, such as medical informatics, mobile phones, anxiety, intervention and prevention, and healthcare quality. From 2015 to 2020, there was a continuation and further development of these earlier keywords, including mobile apps, resilience to stress, public health, and mental health intervention. From 2020 to 2024, the emergence of new keywords significantly decreased, with the focus shifting to intervention methods and new target populations such as university students.

**Figure 11:**
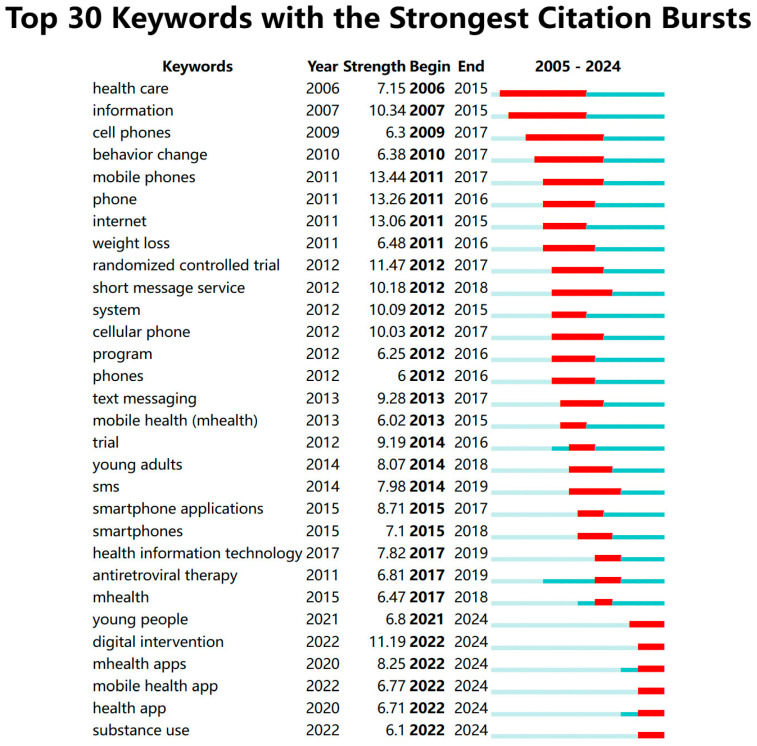
Top 30 Keywords with the Strongest Citation Bursts. Note: Figure 11 shows keyword citation burst patterns in mHealth research (2005–2024). Early bursts (2006–2012) focused on foundational concepts like healthcare and cell phones, with “mobile phones” having the strongest burst (13.44) in 2011. Mid-period (2013–2017) emphasized applications like weight loss and text messaging. Recent bursts (2020–2024) concentrate on digital interventions, young people, and mobile health apps, reflecting the field’s current priorities. This keyword citation burst visualization employs a three-color system to effectively represent temporal patterns in research focus. The light blue background spans the entire study period (2005–2024), providing a consistent reference frame. Overlaid on this, darker blue/teal segments indicate when specific keywords actively appeared in the literature, marking their presence in academic discourse from first emergence to final mention. The striking red sections highlight periods of citation bursts–moments when particular keywords experienced dramatic spikes in scholarly attention.

Keywords such as information, mobile phone, phone, and randomized controlled trial experienced a burst in citations starting from 2011 and continued until 2018, reflecting strong research interest in the early 2010s (Figure 11). From 2015 to 2018, the focus shifted toward applications on smartphones, mobile information technology, platforms, and others. Emerging keywords such as mHealth apps, digital interventions, and weight management began to show citation bursts in 2022, indicating that these areas may be attracting increased research attention in the present and future. Research on smartphone apps has continued for a long period, with keywords evolving from smartphone app to mHealth app, which signifies mHealth apps have become a distinct category. The increase in digital intervention aligns with the growing body of research in digital health. Research on mobile devices, such as smartphones, has remained a constant focus throughout the period. We have also noticed that since 2014, research in mobile health has increasingly focused on young adults, and by 2018, the target audience of mobile health services expanded to include all young people, further incorporating children and adolescents, as highlighted in the previous analysis.

## 4. Discussion

### 4.1. Publishing Trends of mHealth Literature

The rise of mHealth represents a significant breakthrough in the rapid advancement of information technology. mHealth has made healthcare more accessible to a broader audience. The volume of mHealth research published between 2011 and 2020 grew exponentially, indicating that, initially, acceptance was low, user adoption was limited, and progress in the field was relatively slow. As the number of mHealth users gradually expanded, increasing attention from researchers led to a surge in publications. However, starting in 2020, the growth rate began to slow down, with some indications of a gradual decline in the number of publications from 2020 to 2024.

Based on the theory of diffusion of innovation [24], the adoption of mHealth was slow at first, with a gradual increase in users and research. Over time, as more users embraced mHealth, the growth in both usage and academic publications accelerated. However, as the market became saturated and the technology matured, the growth rate began to decelerate, reflecting the typical saturation point observed in the diffusion of innovations. This trend is evident in the slowdown of mHealth publications from 2020 to 2024, signaling that the field is nearing a stage of equilibrium or consolidation.

The number of publications on mHealth has reached a saturation point, but we think that the technology behind mHealth is maturing. In recent years, research in the field has shifted towards mobile apps, driven by the advancement of information technology, with an increasing focus on studies involving digital interventions. This indicates that more and more technologies are being applied in the field. In 2024 alone, over 200 mHealth patents were registered with WIPO, and the mHealth market is projected to grow from USD 82.29 billion in 2024 to USD 261.67 billion by 2029, with a compound annual growth rate (CAGR) of 26.03% during the forecast period [25]. Moreover, the number of studies on randomized controlled trials has gradually decreased, suggesting that the effectiveness of mHealth technologies is widely supported. Therefore, the future of mHealth research looks promising.

Based on our research in Section 3.4 analysis shows that mHealth research experienced a significant peak from 2016 to 2021, a period that coincided with the widespread adoption of mobile devices and the rapid growth of basic mHealth applications. As smartphone research gradually approached saturation, transitioning from rapid growth to replacement cycles, basic health applications became commonplace, no longer generating the novelty that previously sparked high research interest. Research focus has shifted from fundamental mobile health concepts toward more specialized applications and integration with broader healthcare systems, as evidenced by recent citation bursts (2022–2024) in keywords such as “digital intervention”, “mHealth apps”, “mobile health app”, and “substance use”. The transition from exploration to implementation has naturally led to a stabilization in research volume, marking the field’s progression from theoretical development to practical application and optimization. This steady state represents the maturation of the field rather than declining interest.

Regarding funding dynamics, Rock Health’s digital health funding reports indicate that after reaching a peak of USD 29.1 billion in 2021, investment declined to USD 15.3 billion in 2022 and further to approximately USD 10.7 billion in 2023, representing a significant contraction that likely affected research output [26]. This also demonstrates that the research peaks in 2021 and 2022 were likely driven by the high growth resulting from the pandemic, while current interest is returning to normal levels. M&A activity in digital health increased by 35% from 2019 to 2022, with larger companies absorbing smaller innovators, potentially centralizing and streamlining research activities [26]. This serves as another point illustrating how mobile health interest has gradually stabilized into a steady state.

In conclusion, while our bibliometric analysis reveals a stabilization or slight decline in mHealth publication growth from 2020 to 2024, this trend should be interpreted in the context of technology maturation rather than diminishing research interest. Several factors likely contribute to this stabilization: increased focus on implementation rather than proof-of-concept studies, consolidation of research efforts around established technologies, and shifting publication patterns favoring quality over quantity. The post-pandemic normalization of investment and increased M&A activity suggest industry consolidation that centralizes research efforts, potentially yielding fewer but more substantive publications. Citation bursts in specialized terms like “digital intervention” and “mHealth apps” demonstrate evolution toward practical applications. In addressing biases such as citation lag, indexing delays, and limited coverage of interdisciplinary journals, we have made efforts to utilize authoritative databases, combine information from multiple data sources, and incorporate external data from industry reports. However, our treatment of non-English publications could still be strengthened to improve the geographical representation and comprehensiveness of our analysis.

### 4.2. International Trends

The institutions with the highest publication volume have some commonalities. These institutions have been at the forefront of mHealth research, contributing significantly to the development and expansion of mHealth technologies. Their leading positions can be attributed to strong governmental support, substantial investments in healthcare innovation, and collaborations between academic and clinical researchers [27]. These institutions not only lead in terms of publication volume but also influence global trends in mHealth applications, research methodologies, and the integration of digital health solutions into healthcare systems. In some regions, the development of mHealth is often hindered by limitations in public health infrastructure and technology [27]. As technology continues to become more widespread and living standards improve globally, mobile healthcare solutions will gradually expand their reach into these regions, bringing essential medical services to previously underserved populations [28].

### 4.3. An In-Depth Exploration of Research Hotspots

Recent advancements in mobile health technologies have led to three main trends: increasingly effective mental health interventions, the broader application of emerging technologies like artificial intelligence in disease management, and the expanding target population for mobile health services. These trends reflect the growing potential and acceptance of mobile health solutions across diverse groups and healthcare needs.

As digital technologies continue to revolutionize healthcare delivery, mHealth applications are emerging as powerful tools for empowering individuals to take control of their mental well-being. In the field of mHealth, research on the keyword “mental health” has consistently remained a prominent and highly active area of focus. A previous study highlighted the application of mHealth apps in mental health in their bibliometric analysis, showcasing its importance as a major research focus even before the COVID-19 pandemic [29]. Moreover, the number of articles on mental health in the mHealth area has shown no declining trend from 2021 (133 counts) to 2023 (146 counts) (File S1). The keyword “mental health” is not only a significant component of mHealth but also a key focus among leading authors in related fields.

Compared to traditional healthcare models, mHealth can better address patients’ psychological factors because it provides greater accessibility, flexibility, and continuous support through mobile devices. The findings indicate that mHealth, when combined with peer support features and additional emotional support tools like mindfulness activities, is beneficial for adolescents, perinatal women, and marginalized groups, leading to decreased depressive scores and increased satisfaction with accessibility and flexibility [30,31,32]. mHealth also shows a statistically significant reduction in PTSD, depression, and insomnia symptoms and is widely accepted by military personnel [33]. The valuable aspects of mHealth are its ability to overcome spatial limitations and alleviate patients’ psychological and emotional distress with minimal healthcare resources.

mHealth can help address a variety of diseases through digital intervention. mHealth tools, including insulin management applications, wearable blood glucose meters, automated text messages, health diaries, and virtual health coaching, play a multifaceted role in diabetes management by enhancing insulin management, diabetes education, self-management, and prevention [34]. Additionally, in China, there are also apps like WeiSugar and Sugar Nurse that help treat diabetes patients by providing basic services such as blood glucose monitoring and doctors. It is highlighted that digital technology and mHealth have shown potential in delivering behavioral treatment for cardiovascular disease [35]. It is also pointed out that people widely accept mHealth interventions for HIV prevention, particularly through smartphone applications [36,37]. Indeed, the widespread use of mobile technology in the prevention and management of various diseases highlights its potential as a powerful tool in public health and medical care.

We hypothesize that advancements in technology have driven the expansion of the target population for mobile health. The keyword burst has shifted from “young adults” to “young people”, and in recent years, there has been an increase in research focusing on groups such as children, university students, and adolescents [38]. Mobile health is being applied to populations beyond adults, with notable contributions to areas such as polio, adolescent obesity, and pediatric chronic diseases [39,40,41]. This reflects how mobile health technologies are becoming increasingly relevant and beneficial in addressing health challenges across diverse age groups, demonstrating their potential to support a wide range of healthcare needs in younger populations.

The bibliometric analysis of collaboration networks in mHealth research reveals critical implications for policy development. Our findings demonstrate that countries and institutions with robust collaborative ties produce higher-impact research that more effectively influences regulatory frameworks. Notably, interdisciplinary collaborations between technology developers, healthcare providers, and policy experts have yielded the most comprehensive privacy and security guidelines. Furthermore, our network analysis reveals that regions with stronger industry-academic-government collaborative triangles show a more rapid increase in research. These patterns suggest that policymakers should actively promote cross-sector and international research partnerships to develop more effective and culturally appropriate mHealth governance frameworks. By strategically strengthening collaboration networks identified in our study, future policy development can better address the complex challenges of privacy protection, ethical AI implementation, and equitable access across diverse populations.

Health has proven to be a transformative tool in healthcare, particularly in the realm of mental health, where it offers increased accessibility, flexibility, and real-time support. Research in this field has shown consistent growth, especially in areas such as digital interventions, mobile apps, and the integration of peer support and emotional tools. mHealth is not only effectively addressing psychological factors but also enhancing patient self-management for various conditions, such as diabetes and HIV prevention. However, technological advancements have also brought concerns about privacy. mHealth technology also is evolving, with a growing emphasis on younger demographics. The future of mHealth research looks bright, with promising potential for continued innovation and impact on a global scale.

### 4.4. Potential Concerns Regarding AI Applications in Digital Health

The integration of AI technology in digital health has demonstrated a significant impact. The trend reveals important concerns regarding AI acceptance, transparency, ethics, and data privacy. These latent concerns within digital health warrant attention as they may evolve into substantive challenges in the future. Currently, discussions around ethical considerations mainly focus on formulating relevant policies and protecting sensitive data, among other issues.

Health technologies pose multiple privacy and security challenges. These digital systems generate continuous data streams capturing sensitive personal information. Anonymized health data remains vulnerable to re-identification through data linkage techniques [42]. Cybersecurity threats can compromise both patient data and safety. Sensitive health information faces risks of misuse or unauthorized access by third parties [43]. Regulatory frameworks like GDPR and HIPAA attempt to balance necessary data protection with efficient information flow [44]. Digital health technology should be guided by principles of transparency and accountability, ensuring users understand the entire process of data collection and usage to build trust and protect privacy rights [45].

From a data privacy protection perspective, enhancing comprehensive data protection legislation and strengthening privacy safeguards for vulnerable populations engaging with AI-powered mHealth applications, particularly those under criminal legal system supervision, is critically important [46]. It is emphasized that effective digital medicine implementation requires not only robust technical safeguards and regulatory frameworks like GDPR but also addressing the unequal impacts of privacy concerns on vulnerable populations whose limited digital privacy literacy and historical distrust of institutions create additional barriers to healthcare technology adoption [47].

In conclusion, balancing innovation with privacy protection remains a fundamental challenge in digital health. Future success depends on combining technological safeguards with appropriate regulations, enhanced digital literacy, and privacy-by-design approaches that address the needs of diverse populations, particularly those most vulnerable to privacy violations.

### 4.5. Frontier Directions in mHealth Research

Based on the analysis results, we have gained some insights about the possible future development directions of mHealth. These include modern digital and artificial intelligence technologies, such as the Internet of Things, blockchain, VR, and artificial intelligence algorithms.

Digital health technologies have revolutionized healthcare data management through wearable devices, mobile applications, online platforms, and electronic records. These innovations capture, store, and visualize health information, empowering patients, caregivers, and clinicians to better understand and address medical conditions. The transformative potential of these technologies includes enhanced operational efficiency, rapid scalability, near-instantaneous data sharing, and accelerated analytics [48]. The integrated Internet of Things smart patient care system significantly reduced inpatient falls by 88% through effective bed-exit detection, accurate alarm systems, and mobile alerts that enabled healthcare staff to respond efficiently to potential fall situations [49]. Smart devices integrated with the Internet of Medical Things (IoMT) offer transformative potential for primary care by connecting patients and providers through health applications that leverage real-time data monitoring, though significant implementation challenges remain for full clinical integration [50]. mHealth technologies are transforming healthcare through interconnected systems that protect privacy, enhance personalization, and bridge physical distances between patients and providers.

To deal with data privacy, federated learning technology could become a focal point in mobile healthcare due to its ability to protect patients’ personal privacy while enabling collaborative model training across distributed medical data. Federated learning enables multiple healthcare entities to collaboratively train AI models on real-world data while preserving patient privacy, offering a solution to the data accessibility challenges in digital health research without compromising disclosure risk [51]. Federated learning ensures privacy in healthcare metaverse applications but faces challenges of data heterogeneity and security threats, which FedKC addresses through enhanced personalization and robust defense against poisoning attacks [52].

Blockchain technology enhances mobile healthcare data sharing by providing decentralized authentication while preserving privacy through hidden access policies and improving efficiency via offline mechanisms in attribute-based encryption schemes [53]. Blockchain in healthcare will evolve toward interoperable, energy-efficient platforms with specialized use cases in medication verification, clinical trials, and patient-controlled records. Regulatory maturity will drive the adoption of hybrid models using smart contracts and privacy-preserving computation, transforming blockchain from a speculative technology into an essential healthcare infrastructure that enhances trust while remaining largely invisible to end users.

Virtual reality technologies are transforming telehealth for chronic pain management by enabling immersive, interactive patient-physician experiences through advances in biofeedback, haptics, 3D conferencing, and Metaverse environments, despite ongoing implementation challenges [54]. Virtual reality technology, though still limited in application for the physical rehabilitation of cancer patients during chemotherapy, demonstrates unique potential in providing immersive rehabilitation experiences and improving patient treatment engagement [55]. Virtual reality in healthcare enables immersive therapeutic experiences, medical training simulations, and remote patient monitoring while enhancing engagement across various clinical applications from pain management to rehabilitation therapy [48]. Virtual reality will revolutionize healthcare by creating seamless digital spaces where clinicians can conduct remote physical examinations, deliver immersive therapeutic interventions, and enable patient participation in personalized treatment simulations—transforming passive care into active, embodied healing experiences regardless of physical distance.

In silico techniques, including genomic sequencing, pharmacometric modeling, digital health monitoring, and artificial intelligence, are revolutionizing healthcare by enabling precision medicine to deliver truly personalized treatments based on individual genetic, environmental, and lifestyle factors [56]. Digital health technologies are driving the evolution of personalized medicine by enabling sophisticated algorithms to analyze sensor and mobile application data, allowing healthcare providers to customize decisions and treatments to each patient’s unique needs while controlling critical disease parameters.

Artificial intelligence in mHealth revolutionizes patient care through predictive algorithms that analyze real-time data from wearable devices and mobile applications to identify health risks and recommend personalized interventions. These AI-powered systems enhance clinical decision-making by automating routine tasks such as medication management and symptom tracking while enabling more precise remote monitoring that adapts to individual health patterns. Artificial intelligence in mHealth platforms shows promising potential for predicting youth mental health symptoms through smartphone usage patterns, sleep metrics, and physical activity data, though challenges remain in addressing sample size limitations, privacy concerns, and the need for more rigorous evaluation before clinical integration [57].

### 4.6. Comparison with Prior Work

We strived in recent years of publications to perform a bibliometric analysis to understand the latest developments in mobile healthcare, which provides a multi-dimensional analysis. It indicates the future direction of mobile healthcare from the perspective of technological trends while considering factors such as target populations, artificial intelligence, and other relevant aspects.

### 4.7. Limitations

To our knowledge, this study represents the most recent bibliometric analysis of mHealth research. However, there are several limitations to our approach. First, although we used a strategy aimed at capturing mHealth-related studies accurately, some literature unavoidably missed out. Secondly, we did not use some extended keywords for clustering analysis, which may have resulted in certain subtle trends being missed or not fully captured. Thirdly, our search was restricted to English-language publications, which may limit the scope of our findings. The data for this analysis were exclusively sourced from the Web of Science, without considering other significant databases like Scopus and PubMed. Although the Web of Science is a large and reliable database, many relevant studies may only be indexed in other databases, potentially influencing the outcome of our study. The Chinese Science Citation Database also contains numerous papers published in Chinese. Therefore, to improve the accuracy and comprehensiveness of future research, it would be beneficial to incorporate additional databases and languages.

## 5. Conclusions

This study reveals the latest research trends and hotspots in mobile health, as well as the current state of the industry. As mentioned earlier, mobile health has shown immense potential in various aspects of our lives in recent years, with an increasing number of new technologies being applied to assist various patients. However, the development of mobile health is still constrained by certain technological limitations, and its progress is slower in developing countries. Therefore, we recommend that researchers in this field continue to explore these issues to further advance mobile health. We also hope that the findings of this study will provide valuable guidance for future mobile health research.

## Figures and Tables

**Table 1:** Distribution of publications from different countries and institutions about mHealth.

Institution	Count	Centrality ^a^	Year
University of California System	248	0.09	2011
Harvard University	225	0.05	2012
University of London	208	0.04	2013
Harvard Medical School	144	0.03	2012
Johns Hopkins University	130	0.04	2013
University of Sydney	130	0.02	2014
State University System of Florida	128	0.1	2014
University of Toronto	126	0.04	2013
Pennsylvania Commonwealth System of Higher Education (PCSHE)	106	0	2013

^a^: Centrality measures the importance or influence of an institution within a network, indicating its ability to connect and bridge with other nodes effectively.

**Figure A1:**
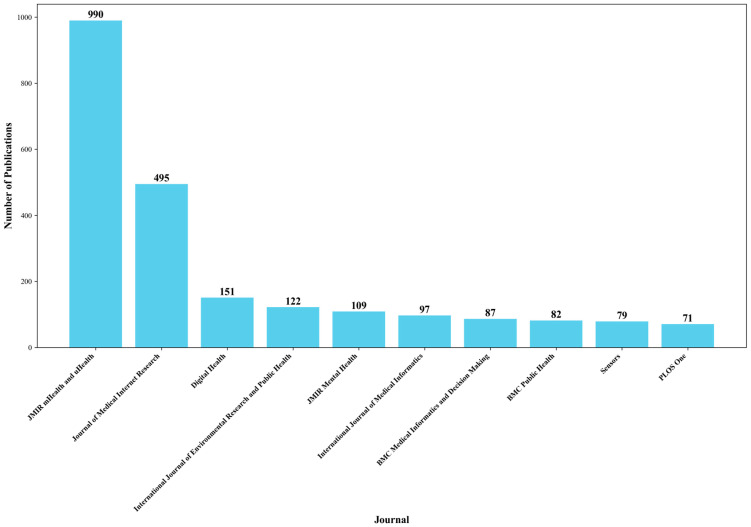
Number of publications per journal from 2005 to 2024.

**Figure A2:**
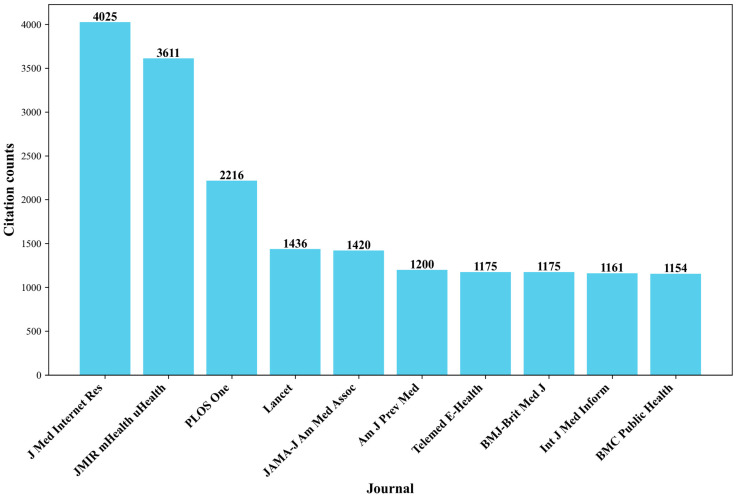
Top 10 journals by citation count.

**Figure A3:**
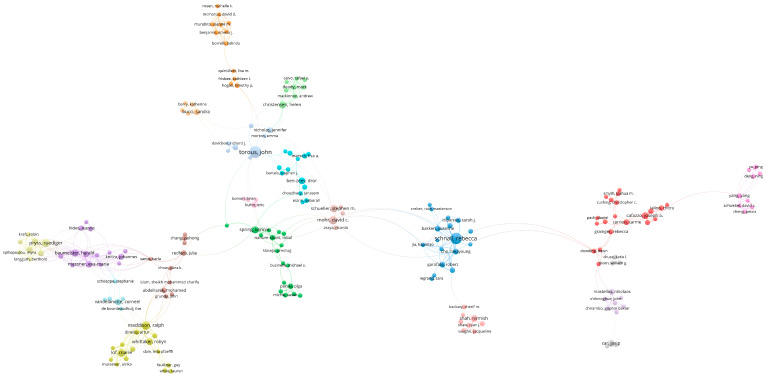
Keyword clustering analysis of mHealth research using CiteSpace. Note: Node colors represent different thematic clusters labeled with their dominant topics, node size reflects the importance or frequency of the terms, and edges show relationships or co-occurrence between terms within and across clusters.

## Data Availability

The original contributions presented in this study are included in the article/Supplementary Materials. Further inquiries can be directed to the corresponding author.

## Supplementary Materials

The following supporting information can be downloaded at: https://www.mdpi.com/article/10.3390/healthcare13070740/s1, File S1: The raw data collected during the experimental process includes publish counts, digital health, institutions, countries, keywords, co-cited journals, published journals, authors, and co-cited data.

## Abbreviations

The following abbreviations are used in this manuscript:

mHealth	mobile health
AI	Artificial Intelligence
WOS	Web of Science database
GDPR	General Data Protection Regulation
HDAA	Health Insurance Portability and Accountability Act

## Appendix A. Journals and Co-Cited Academic Journals

A total of 6093 articles related to mHealth were published in 1171 journals. The statistical chart of the top 10 journals is as follows (Figure A1). The “JMIR mHealth and Uhealth” journal has the highest number of articles with 4025, followed by the “The Journal of Medical Internet Research “with 495 articles. The impact factors of “JMIR mHealth and uhealth” and “The Journal of Medical Internet Research” are 5.4 and 5.8, respectively, which are also the highest among the top ten journals, indicating that they may hold significant positions in the field of mHealth. “JMIR mHealth and uHealth” focuses on health and biomedical applications in mobile and tablet computing, pervasive and ubiquitous computing, wearable computing and domotics, which are central to mHealth”. The Journal of Medical Internet Research” reports the design and evaluation of health innovations and emerging technologies, including emerging technologies related to digital health, telemedicine, and health applications, which addresses a broader spectrum of digital health innovations.

To better understand the core journals in the field of mHealth, we performed a co-citation analysis. Co-citation analysis aims to measure the degree of association between journals. The impact of a journal depends on its citation frequency, which reflects the journal’s influence in a specific research field. Among the co-cited journals, the top 10 journals (Figure A2 and Table A1) have all been cited more than 1100 times. “Journal of Medical Internet Research” has the highest number of citations with 4025, followed by “JMIR mHealth and uHealth”, “Plots One”, “Lancet”, “Jama-Journal of the American Medical Association”, and “British Medical Journal”. “Lancet” has the highest impact factor, which is 168.9, followed by “British Medical Journal” with 105.7. Among them, “JMIR mHealth and uHealth” is the most focused on mHealth, publishing research on mobile technologies directly related to healthcare. Other journals like “PLOS One”, “Lancet”, “Jama-Journal of the American Medical Association”, and “British Medical Journal” cover mHealth within broader medical and healthcare contexts, often evaluating the clinical effectiveness and impact of mHealth technologies on patient care, health outcomes, and global health. This suggests that the references for mHealth extend beyond the domain of mHealth itself and are also significantly supported by established traditional journals.

**Table A1:** Distribution of co-cited journals.

Journal	Count	Year	Impact Factor
J MED INTERNET RES	4025	2011	5.4
JMIR MHEALTH UHEALTH	3611	2014	5.4
PLOS ONE	2216	2013	2.9
LANCET	1436	2011	168.9
JAMA-J AM MED ASSOC	1420	2011	63.1
BMJ-BRIT MED J	1200	2010	105.7
AM J PREV MED	1175	2005	4.3
TELEMED E-HEALTH	1175	2012	2.8
INT J MED INFORM	1161	2006	3.7
BMC PUBLIC HEALTH	1154	2012	3.5

## Appendix B. Authors and Co-Cited Authors

The top ten authors in the field of mHealth are presented in Table A2. Schnall R and Torous J have published the most articles, with both reaching 41 papers, followed by Bousquet J with 32 publications. Overall, Torous J and Schnall R have the highest number of publications and have formed their respective collaboration networks. John Torous, MD, is a highly influential researcher in the fields of Psychiatry and Psychology, recognized for his work in Digital Psychiatry and mHealth. Among them, Dr. Torous is known for his pioneering research on the application of digital health technologies in mental health care and has received the highly cited researcher award in 2022 and 2023 for his impactful contributions to the field. Rebecca Schnall is a distinguished scholar at Columbia University School of Nursing, where she contributes significantly to the fields of Health Care Sciences, Public Health, Medical Informatics, Nursing, and Computer Science. Jean Bousquet is also a globally renowned expert in respiratory health, having chaired GINA, ARIA, and GARD while publishing over 1000 papers. His groundbreaking work on allergy mechanisms, severe asthma, and the digital transformation of health has solidified his status as the most influential author in asthma research. In conclusion, they apply mHealth tools to different areas, but both aim to make healthcare more accessible, personalized, and effective through the use of technology, showcasing the broad applicability of mHealth across medical domains.

There is a network of communication and collaboration among authors in this research field (Figure A3). Two or more authors who are cited together are referred to as co-cited authors. In Figure A3, each node represents an author, larger nodes signify a higher number of published articles or citation frequency, and thicker lines indicate closer collaboration between authors.

Among the co-cited authors shown in Table A3, Stoyanov SR leads with an impressive record count of 459, reflecting significant influence and recognition in their field. Following closely is Michie S, with 423 records, indicating substantial contributions to scholarly discourse. Eysenbach G also garners notable attention with 361 co-citations. Meanwhile, Moher D, with 368 records, and Free C, with 351 records, both command a strong presence, highlighting their academic impact. Both Stoyanov SR and Michie S focus on the intersection of smartphone applications and mental health, emphasizing the transformative potential of technology in mental health care. The top co-cited authors show the significant collaborative impact and influence in the field of mHealth research, with their work often serving as foundational references for advancing digital health innovations.

**Table A2:** Top ten authors in the field of mHealth based on publication count.

Authors	Record Count	Percent
Schnall R	41	0.67%
Torous J	41	0.67%
Bousquet J	33	0.54%
Lee S	26	0.43%
Kim Y	26	0.43%
Pryss R	24	0.39%
Lee J	24	0.39%
Mohr DC	24	0.39%
Kim H	22	0.36%

**Table A3:** Top six co-cited authors in mHealth research.

Authors	Record Count
Stoyanov SR	459
Michie S	423
Eysenbach G	368
Moher D	366
Free C	351
Torous J	349
